# Neurophysiological Endophenotypes, CNS Disinhibition, and Risk for Alcohol Dependence and Related Disorders

**DOI:** 10.1100/tsw.2007.203

**Published:** 2007-11-02

**Authors:** Bernice Porjesz, Madhavi Rangaswamy

**Affiliations:** Henri Begleiter Neurodynamics Laboratory, SUNY Downstate Medical Center, Brooklyn, NY, USA

**Keywords:** endophenotypes, EEG, event-related potentials

## Abstract

Biological endophenotypes are more proximal to gene function than psychiatric diagnosis, providing a powerful strategy in searching for genes in psychiatric disorders. These intermediate phenotypes identify both affected and unaffected members of an affected family, including offspring at risk, providing a more direct connection with underlying biological vulnerability. The Collaborative Study on the Genetics of Alcoholism (COGA) has employed heritable neurophysiological features (i.e., brain oscillations) as endophenotypes, making it possible to identify susceptibility genes that may be difficult to detect with diagnosis alone. We found significant linkage and association between brain oscillations and genes involved with inhibitory neural networks (e.g.,* GABRA2, CHRM2*), including frontal networks that are deficient in individuals with alcohol dependence, impulsivity, and related disinhibitory disorders. We reported significant linkage and linkage disequilibrium for the beta frequency of the EEG and *GABRA2*, a *GABA_A_* receptor gene on chromosome 4, which we found is also associated with diagnosis of alcohol dependence and related disorders. More recently, we found significant linkage and association with *GABRA2* and interhemispheric theta coherence. We also reported significant linkage and linkage disequilibrium between the theta and delta event-related oscillations underlying P3 to target stimuli and *GABRA2*, a cholinergic muscarinic receptor gene on chromosome 7, which we found is also associated with diagnosis of alcohol dependence and related disorders. Thus, the identification of genes important for the expression of the endophenotypes (brain oscillations) helps when identifying genes that increase the susceptibility for risk of alcohol dependence and related disorders. These findings underscore the utility of quantitative neurophysiological endophenotypes in the study of the genetics of complex disorders. We will present our recent genetic findings related to brain oscillations and Central Nervous System (CNS) disinhibition.

Alcoholism is a common, complex (non-Mendelian) disorder with contributions from both genetic and environmental influences and their interactions. As psychiatric diagnosis is dichotomous (an individual is either affected or unaffected), it is difficult to use diagnosis as the sole phenotype when studying the genetics of complex disorders, such as alcoholism. It has been suggested that, ideally, molecular genetic studies should not be performed on psychiatric diagnoses alone, which reflect distal and variable effects of genes, but on quantitative neurobiological measures or markers that reflect more proximal effects of genes involved in the genetic predisposition for developing psychiatric disorders[1]. These quantitative biological markers (endophenotypes or intermediate phenotypes) serve as covariates that correlate with the main trait of interest (diagnosis) and serve to better define that trait or its underlying genetic mechanism[2,3,4]. The advantages of using quantitative neurobiological measures of risk as endophenotypes in the search for genes involved in complex disorders are that they are closer to gene action involved in the predisposition for the disorder, they are genetically simpler than clinical endpoints, and quantitative traits provide more power to localize and characterize disease susceptibility genes[5].

Recent evidence suggests that alcohol dependence is part of a spectrum of disinhibitory disorders, which include externalizing and substance use disorders. Many of the same genetic risk factors underlie these disinhibitory co-occurring disorders and can be explained by a small number of common underlying genetic factors[6]. Genetic studies of neuroelectric endophenotypes (brain oscillations, such as EEG and event-related potentials [ERPs], including the P3 component) provide a powerful strategy for identifying underlying susceptibility genes for developing these disinhibitory disorders.

## BRAIN OSCILLATIONS AS ENDOPHENOTYPES

Brain oscillations provide a rich source of potentially useful endophenotypes for psychiatric genetics, as they represent important correlates of human information processing and are associated with fundamental processes of perception to cognition. These oscillations are dynamic indices of the millisecond-by-millisecond balance between excitation and inhibition in brain neural networks. Brain oscillations reflect ensembles of neurons firing in synchrony and represent the basic mechanism of neural communication. High-frequency (beta, 12–28 Hz; gamma, 28+ Hz) synchronizations are involved in short-range communication, while low frequencies (delta, 1–3 Hz; theta, 4–7 Hz; alpha, 8–12 Hz) are involved in longer-range communication between brain areas[7]. Brain oscillations represent important correlates of human information processing and cognition. They represent traits less complex and more proximal to gene function than either diagnostic labels or traditional cognitive measures. Therefore, these oscillations may be utilized as phenotypes of cognition, and as valuable tools for the understanding of some complex genetic disorders. It should be noted that brain oscillations meet criteria for endophenotypes and are highly heritable: delta 76%, theta 89%, alpha 89%, beta 86%[8].

In the Collaborative Study on the Genetics of Alcoholism (COGA), we have successfully used brain oscillations as endophenotypes in the search for genes involved in alcohol dependence and related disorders. We have focused on resting eyes-closed EEG, as well as oscillations recorded during sensory and cognitive tasks (ERPs) that differentiate between alcoholics and controls, and between high-risk offspring and controls.

## RESTING EEG BETA POWER: *GABRA2* GENE

Increased beta power in the resting EEG of alcoholics has been well documented[9,10,11,12,13]. Several studies also report increased resting beta power in offspring of alcoholics[14,15,16], and it has been reported at frontal leads in those who also have a diagnosis of antisocial personality disorder (ASP)[17]. Increased resting beta power has also been reported in a large sample of offspring of alcoholics at high risk from the COGA project[16]. As it is already observed in offspring before the onset of alcohol dependence, this increased beta antecedes the development of alcoholism and is considered to be a trait rather than a state measure.

Significant linkage and linkage disequilibrium between the EEG beta frequency and a $GABA_A$ receptor gene on chromosome 4 has been reported by COGA[18] (Fig. 1). Subsequent SNP (single nucleotide polymorphism) analysis in COGA indicated that the same *GABRA2* gene associated with the EEG beta endophenotype is associated with DSM-IV diagnosis of alcohol dependence[19], substance dependence[20], ASP[21], and childhood conduct disorder[22]. The association between *GABRA2* and alcohol dependence has been replicated by a number of groups throughout the world[23,24,25,26].

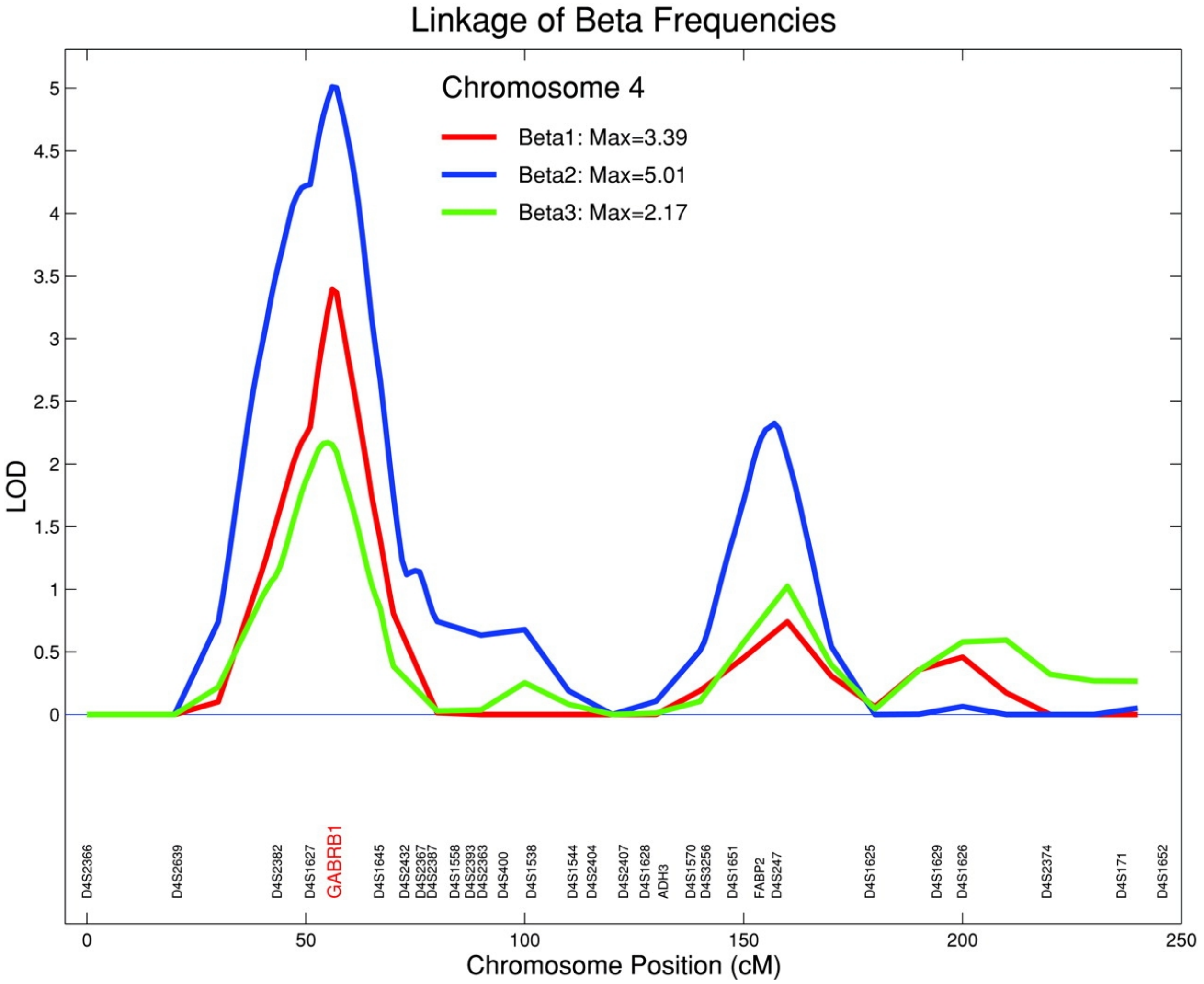

**FIGURE 1**. Linkage results of resting EEG beta band power for beta 1 (12.5–16 Hz), beta 2 (16.5–20 Hz), and beta 3 (20.5–28 Hz) on chromosome 4 at a cluster of $GABA_A$ receptor genes. (N = 1553 individuals from 250 families.)

One of the important mechanisms of the beta rhythm generation is based on properties of inhibitory circuits, and the balance between networks of excitatory pyramidal cells and inhibitory interneurons that involve $GABA_A$ action as the pacemakers[27]. This suggests that variations in the *GABRA2* gene affect the inhibitory tone and, thus, affect the level of neural excitability, which in turn affects the predisposition to develop alcohol dependence and related disorders. As alcoholics and offspring at high risk manifest increased beta activity, this indicates an imbalance between excitation-inhibition (CNS disinhibition). This provides a biological hypothesis relating the underlying CNS disinhibition to the genetic risk for alcohol dependence and related disorders[28]. The involvement of the GABAergic system in alcoholism is supported by neuroimaging studies, which report specific deficits in GABA benzodiazepine receptors in the brains of alcoholics[29,30,31] and individuals at risk[32]. Taken together, these findings suggest GABA deficits in the brains of alcoholics, and individuals at risk may account for their lack of CNS inhibition (hyperexcitability) and may be involved in the predisposition to develop alcoholism.

## INTERHEMISPHERIC THETA COHERENCE: *GABRA2* AND *CHRM2* GENES

Another EEG measure that was examined by COGA is EEG coherence, a measure of cortical synchronization in neural networks, indexing communication in populations of neurons. Recent studies have implicated involvement of thalamocortical circuitry in the theta frequency coherence[33]. We recently observed that alcoholics in the resting state have higher coherence in most frequency bands overall when compared to age-matched controls. Alcoholics manifest significant increases in resting EEG interhemispheric high theta (6–7 Hz) coherence, particularly posteriorly at parietal-occipital and centro-parietal regions[34]. This increased coherence observed in alcoholics is indicative of altered thalamo- and cortico-cortical functional connectivity.

Highly significant linkage was found in COGA on chromosome 4 for the same *GABRA2* gene and high theta coherence at parietal-occipital leads[35] (Fig. 2). Family-based association analyses with the cluster of $GABA_A$ receptor genes under this linkage peak revealed strong association with a large number of SNPs (several at $p < 0.001$) genotyped in *GABRA2* for the Caucasian-only subset. There was no significant association with other genes in the $GABA_A$ cluster on chromosome 4. These results suggest that *GABRA2* might influence susceptibility to alcohol and drug dependence not just by modulating level of neural excitation, but also by influencing functional connectivity of interhemispheric networks.

In addition, significant linkage and association for the interhemispheric high theta coherence centro-parietal phenotype was found with a muscarinic acetylcholine receptor (M2) gene (*CHRM2*) on chromosome 7. Significant linkage and association for evoked theta band responses at the same *CHRM2* gene have been previously reported[36,37] (see below).

Both the GABAergic and cholinergic systems interact significantly in the functions of local inhibitory circuits, thus affecting network functions. Increased strength of GABAergic inhibition has been reported to be a likely mechanism underlying the impaired synaptic plasticity observed with M2 knockout mice. These mice demonstrate impaired behavioral flexibility and memory deficits[38]. In a recent study examining muscarinic control of long-range cortical GABAergic inhibition, the authors suggested that M2 receptor activation produces a presynaptic inhibition of GABA release by long-range inhibitory neurons of the perirhinal cortex projecting to the entorhinal cortex[39]. These networks have been shown to be highly relevant to memory-related processes.

## THETA AND DELTA EVENT-RELATED OSCILLATIONS UNDERLYING P3: *CHRM2* AND *GRM8* GENES

The P3 component has repeatedly been reported to be reduced in alcoholics and in offspring at high risk[40,41], providing a good phenotype for genetic studies. Linkage studies have been performed on P3 amplitude[41]. However, the P3 component is not a unitary phenomenon, but emanates from multiple sources in the brain with contributions from frontal cortex (including anterior cingulate) and hippocampus[42,43,44,45]. Several studies have demonstrated that P3 responses are primarily the outcome of theta (4–7 Hz) and delta (1–3 Hz) oscillations elicited during cognitive processing of stimuli[46,47,48,49,50,51], with delta oscillations more concentrated in the posterior region, while theta is more centered in the fronto-central region[50]; theta oscillations also contribute strongly to N2 components. Reciprocal synchronization has been observed in the theta range between hippocampus and frontal and parietal regions in the brain during attentional tasks[7].

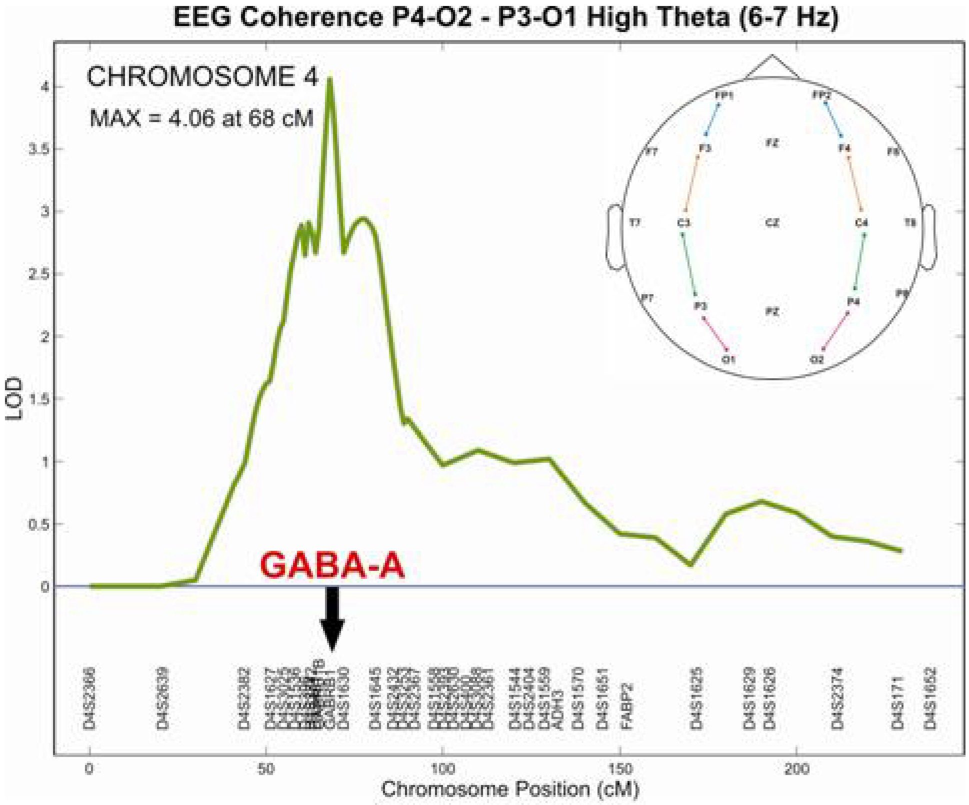

**FIGURE 2.** Linkage results of resting EEG of high theta band (6–7 Hz) interhemispheric coherence at parieto-occipital bipolar pairs of electrodes (P4-O2—P3-O1) on chromosome 4, indicating a significant linkage peak over the cluster of $GABA_A$ receptor genes. (N = 1312 individuals from 251 families.) Inset: head map of electrode pairs (P4-O2—P3-O1 in red).

Therefore, COGA genetic analyses were performed on the event-related oscillations (EROs) underlying the P3, namely the delta and theta EROs. Theta and delta oscillations underlying P3 are related to different cognitive functions: theta is associated with memory processes and attention, and involves fronto-limbic or cortico-hippocampal interactions, and is taken as an index of frontal processing; delta is related to signal detection and decision making, and is generated by cortico-cortical interactions and is prominent after the target stimuli.

It has recently been reported that both delta and theta EROs are reduced in alcoholics compared to controls to target stimuli during a visual oddball paradigm[52] (Fig. 3.) These findings are most significant anteriorly for theta and posteriorly for delta. These theta and delta EROs underlying P3 to targets are already reduced in adolescent offspring at risk, and are more sensitive than P3 amplitude in discriminating between high- and low-risk offspring[53] (Fig. 3).

Significant linkage and association for frontal theta oscillations and the *CHRM2* gene on chromosome 7 (measured genotype and QPDT) were found; association for parietal-occipital delta EROs was also reported[36,37] (Fig. 4). These findings implicate the possible role of *CHRM2* in the generation and modulation of evoked oscillations. Theta and delta EROs depend on the level of acetylcholine (muscarinic activation). M2 receptors inhibit presynaptic release of acetycholine, leading to inhibition of irrelevant networks. Muscarinic receptors influence many effects of acetylcholine in the central and peripheral nervous system, and, hence, are expected to have a direct influence on P3 generation[54]. It is

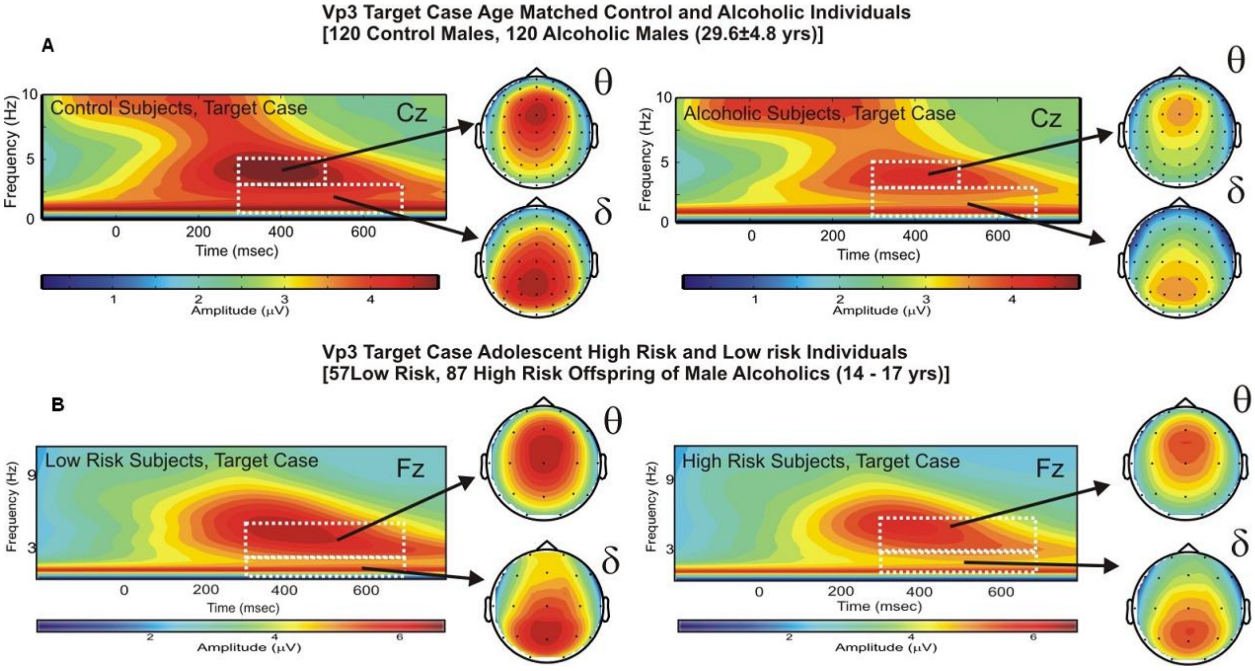

**FIGURE 3**. **(A)** S-transformed time-frequency plots for the target condition at Cz electrode (central midline) in 120 alcoholic (right panel) and 120 control (left panel) subjects, z-scored for each frequency. The head plots of the time-frequency regions of interest (TFROIs) indicate that the theta band (4–5 Hz) power during target processing (300–500 msec) has a more frontal topography while the delta band (1–3 Hz) power has a more posterior topography when P3 is maximum (350–700 msec). Note that alcoholics have weaker responses in both the theta and delta bands during P3 production to target stimuli. **(B)** S-transformed theta (4–7 Hz) and delta (1–3 Hz) band time-frequency plots for the target condition in visual oddball task in 87 high-risk (right panel) and 57 matched low-risk (left panel) adolescent subjects. Plots represent distributions at Fz electrode and are z-scored for each frequency. Head plots for the TFROI that extends from 300 to 700 msec for each band (corresponds to the average temporal range of P3 wave) reveals a frontal maxima for theta band power and a parietal maxima for delta band power in adolescents.

proposed that the genetic underpinnings of evoked oscillations are likely to stem from regulatory genes that control the neurochemical processes of the brain and, therefore, influence neural function. Moreover, the cholinergic muscarinic genes have a major role in memory and cognition[55,56]. The results strongly support the role of acetylcholine in the generation of N2 (theta oscillations) and in the P3 component (delta and theta oscillations). The function of acetylcholine has been demonstrated with regard to stimulus significance[57], selective attention[58], and P3 generation[59]. Administration of cholinergic agonists and antagonists have yielded modified memory performance, and modified P3 amplitude in humans[60,61,62]. *In vitro* administration of moderate amounts of the muscarinic agonist carbachol in the rat hippocampus induces synchronized delta oscillations, whereas higher concentrations produced short episodes of theta oscillations, and that carbachol-induced delta rhythms were not observed concurrent with carbachol-theta[63,64].

Recent evidence from the COGA project indicates that the *CHRM2* gene is not only associated with brain oscillations, but also clinical diagnosis; significant linkage and association were reported for the *CHRM2* gene, and a diagnosis of alcohol dependence and depression as well as a spectrum of externalizing disorders in the COGA sample[65,66]. Thus genes important for the expression of the endophenotype (brain oscillations) help in identification of genes that increase the susceptibility for risk of alcohol dependence and related disorders.

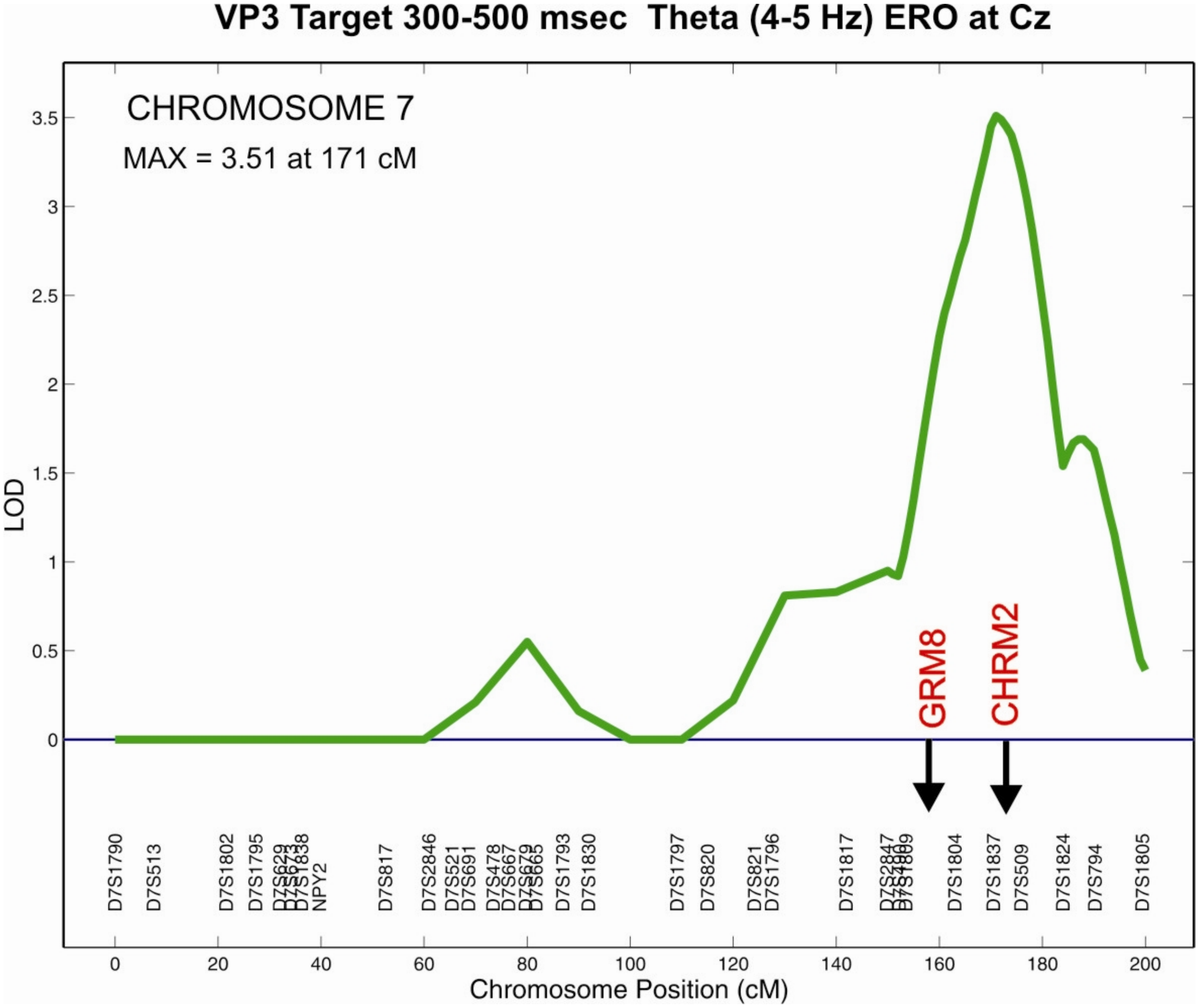

**FIGURE 4**. Linkage result of the target case visual central midline theta (4–5 Hz) band power (between 300–700 msec, when P3 is maximum) on chromosome 7. Maximum LOD scores are situated near the location of a cholinergic muscarinic receptor gene *CHRM2* and a glutamate receptor gene (*GRM8*). The dataset consists of 1337 individuals from 253 families.

Under the same linkage peak on chromosome 7 (Fig. 4) is a glutamate receptor (*GRM8)* gene; family-based association analyses of theta EROs revealed significant associations with several SNPs and theta EROs to target stimuli at frontal, central, and parietal regions[67]. An interesting finding is that several SNPs were also significant for diagnosis of alcohol dependence using ICD-10 diagnostic criteria. *GRM8* modulates glutamatergic transmission in the brain by inhibiting glutamate release at the synapse. The three major neurochemical substrates contributing to theta and delta rhythms and P3 involve strong GABAergic, cholinergic, and glutamatergic system interactions[54].

## IMPLICATIONS: BEHAVIORAL AND CNS DISINHIBITION

The low visual P3 amplitude is not specific to risk of alcohol dependence, but is characteristic of many disinhibitory conditions, such as substance abuse, ASP, conduct disorder, attention deficit hyperactivity disorder (ADHD)[41]. Alcohol dependence is a disorder of disinhibition characterized by disturbed impulse regulation (i.e., the inability to terminate inappropriate behavior). These traits are not unique to alcoholism, but are fundamental to other psychiatric disorders. Clinical manifestations of disinhibition are impulsivity, alcohol dependence, drug dependence, conduct disorder, oppositional disorder, and ADHD. There is evidence that they're a common genetic diathesis. Kendler and coworkers[6] examined the genetic and environmental influences on internalizing and externalizing disorders, and reported that the single most important factor underlying externalizing disorders (alcohol dependence, substance dependence, ASP, conduct disorder) is a common underlying genetic liability involving impulse control. Recently, it was found that alcoholics show an increased level of impulsivity, as measured by the Barratt Impulsivity Scale, and that there is a significant negative correlation between visual P3 amplitude and impulsivity score[68]. Alcoholics and highly impulsive subjects, regardless of diagnosis, showed significantly reduced activation in frontal brain regions (anterior cingulate, cingulate gyrus, medial gyrus, superior frontal gyrus) with low-resolution electromagnetic tomography (LORETA). The genetically influenced differences in susceptibility involve neural disinhibition and impulsivity, which involves frontal lobe functions that influences a range of outcomes, including externalizing and mood disorders, alcoholism, and abuse of other substances[41].

## CONCLUSION

Alcohol dependence and related disorders result from a complex interaction of changing genetic and environmental liabilities across development, with greater genetic loading for early-onset disorders. The use of quantitative brain oscillations as endophenotypes provides the power to more easily localize and characterize disease susceptibility genes than diagnostic categories. The utility of electrophysiological measures as endophenotypes for the study of genetic risk of disinhibitory disorders, including alcoholism, is very promising. While the endophenotype approach is not a new idea, it is an approach whose time has come. Recent advances in SNP-chip technology in molecular genetics, as well as novel statistical genetic techniques and computational power, have made this approach very tenable in the near future. Once genes are identified, risk genotypes and haplotypes can be used in prospective studies of young individuals and can lead to prevention initiatives. These studies are currently underway in the COGA project.

## ACKNOWLEDGMENTS

The Collaborative Study on the Genetics of Alcoholism (COGA), Co-Principal Investigators B. Porjesz, V. Hesselbrock, H. Edenberg, L. Bierut, includes nine different centers where data collection, analysis, and storage take place. The nine sites and Principal Investigators and Co-Investigators are University of Connecticut (V. Hesselbrock), Indiana University (H.J. Edenberg, J. Nurnberger, Jr., P.M. Conneally, T. Foroud), University of Iowa (S. Kuperman, R. Crowe), SUNY Downstate (B. Porjesz), Washington University in St. Louis (L. Bierut, A. Goate, J. Rice), University of California at San Diego (M. Schuckit), Howard University (R. Taylor), Rutgers University (J. Tischfield), Southwest Foundation (L. Almasy). Zhaoxia Ren serves as the NIAAA Staff Collaborator. This national collaborative study is supported by the NIH Grant U10AA008401 from the National Institute on Alcohol Abuse and Alcoholism (NIAAA) and the National Institute on Drug Abuse (NIDA). In memory of Henri Begleiter and Theodore Reich, Principal and Co-Principal Investigators of COGA since its inception; we are indebted to their leadership in the establishment and nurturing of COGA, and acknowledge with great admiration their seminal scientific contributions to the field.

The authors are grateful to the valuable technical assistance of Kevin Jones, Andrew Chen, Chella Kamarajan, Arthur Stimus, and Carlene Haynes.
